# Acute Dilatation of the Stomach

**Published:** 1901-11-02

**Authors:** 


### ACUTE DILATATION OF THE STOMACH.

At the last meeting of the royal Medical and Chirurgical Society, Dr. Campbell Thomson read a paper on Acute Dilatation of the Stomach. This condition, he said, is characterised by its sudden onset, by the vomiting of enormous quantities of fluid, and by very severe general symptoms, which in the recorded cases have always ended fatally within a few days after the first onset of the disease. The condition was first described by Hilton Fagge, and since then a case has been recorded by Mr. Henry Morris, but beyond these the subject does not appear to have attracted much attention. Dr. Campbell Thomson had, however, made post-mortem examinations upon four cases in which death was immediately due to this condition, and he believed that the disease might not be so rare as has generally been supposed, the less serious cases occurring in the course of severe illnesses. Acute dilatation may arise without any apparent cause whatever, the patient being, as far as one can tell, in ordinary health up to the time of the onset of acute symptoms, although in some of the cases there may have been some gradual enlargement of the stomach for some time beforehand, which has been succeeded in the end by sudden symptoms of great severity. Again, in many cases, some other morbid condition is found in addition to the dilatation of the stomach, and in other instances this condition has appeared to follow immediately upon some surgical operation. In another group of cases the ingestion of a large quantity of badly masticated food has appeared, in debilitated subjects, to have been the exciting cause. As to the symptoms of the condition, there is great swelling of the abdomen, filling chiefly the left half and the lower part of the abdomen, the right hypochondrium sometimes appearing to be flattened. Peristaltic waves of contraction do not seem ever to have been observed. Vomiting is a constant symptom, and large quantities of greenish fluid are usually brought up. The urine is generally scanty and is almost entirely suppressed for 24 hours before death. The general symptoms are those of collapse, the pulse is small and very rapid, the respirations are frequent, and the temperature is low, usually subnormal. After death the appearance of the stomach is very characteristic. It is like a tightly distended cylinder, shaped like a **V** with one limb shorter than the other, the angle between the two limbs formed by the lesser curvature being a very sharp one. The intestines are usually collapsed, and appear as if they had been compressed by the dilated stomach. It may be surmised that the immediate cause of the dilatation is some disturbance of the nervous system which gives rise to paralysis of the muscular walls of the stomach, but, as was shown by the diverse views which were set out in the discussion on the paper, the whole subject is evidently involved in much obscurity.

